# Relationship between training status and stress response in Chinese college student-athletes: chain mediation between sport performance strategies and coping styles

**DOI:** 10.3389/fpsyg.2025.1597539

**Published:** 2025-07-09

**Authors:** Jia Gao, Jun Xiang, Zhongren Hou, Hankun Liu

**Affiliations:** School of Physical Education and Health, Zhaoqing University, Zhaoqing, China

**Keywords:** training status, stress response, sport performance strategies, coping styles, collegiate student-athletes

## Abstract

**Aim:**

The stress response is recognized in sport psychology as a complex physiological and psychological reaction elicited by the human body when confronted with challenges or threats. It remains a focal issue in research on athletes’ training status and sport performance.

**Objective:**

This study aimed to examine the effects of training status on the stress response of Chinese college student-athletes and to verify the mediating roles of sport performance strategies and coping styles.

**Methods:**

A total of 797 Chinese college student-athletes were assessed using the Training Status Scale, Stress Response Scale, Sports Performance Strategy Scale, and Coping Style Scale.

**Results:**

(1) Significant differences were observed in training status and sport performance strategy across age, gender, and sport level (*p* < 0.05); significant differences in stress response were found for age and sport level (*p* < 0.05), but not for gender (*p* > 0.05); and significant differences in coping styles were found for sport level (*p* < 0.05), with no significant differences for gender or age (*p* > 0.05). Male athletes had higher mean scores than female athletes in training status, stress response, and sport performance strategy, while both genders scored similarly in coping style. (2) Training status was significantly negatively correlated with stress response (r = −0.679, *p* < 0.001), and had a direct negative effect on stress response (*β* = −0.237, t = −13.539, *p* < 0.001). Additionally, training status positively predicted sport performance strategy (*β* = 0.019, t = 10.211, *p* < 0.001) and coping style (*β* = 0.131, t = 3.495, *p* < 0.001); sport performance strategy significantly predicted coping style (*β* = −0.442, t = −5.879, *p* < 0.001) and stress response (*β* = 0.371, t = 29.986, *p* < 0.001); coping style significantly and positively predicted stress response (*β* = −0.055, t = −1.435, *p* < 0.001). (3) Sport performance strategies and coping styles played significant mediating roles between training status and stress response, accounting for 54.33% of the total effect. Specifically, the mediating effect of sport performance strategy was 10.79%, coping style was 32.37%, and the chain mediation of both was 11.18%.

**Conclusion:**

Training status is a significant predictor of sport performance strategies, coping styles, and stress responses among college student-athletes. Moreover, sport performance strategies and coping styles mediate the relationship between training status and stress response. These findings are valuable for enhancing training status, stress response, sport performance, and coping styles in collegiate student-athletes, and provide a theoretical foundation for intervention development. However, limitations include the specificity of the sample and reliance on self-reported data. Future research should expand the sample scope and size, and employ multiple assessment methods and instruments to validate these findings.

## 1 Introduction

In the field of sport psychology, there is growing recognition of the intricate relationship between athletes’stress responses and their overall health (Nuetzel, 2023; Liu et al., 2021). Research has shown that the stress response is a complex physiological and psychological reaction that occurs when an individual is confronted with a challenge or threat. Commonly referred to as the “fight or flight” response, this adaptive mechanism is designed to mobilize resources and prepare the body to cope with immediate stressors (Zeng and Cui, 2022). During a stress response, the body releases hormones such as adrenaline and cortisol, triggering a cascade of changes including increased heart rate, heightened alertness, and the redirection of energy to essential functions. For athletes, who frequently encounter the pressures of training, competition, and other stressors, stress responses can vary in intensity. While these responses may be beneficial in acute situations, chronic activation of the stress response can have detrimental effects on both physical and mental health (Tian et al., 2011). This is particularly true for collegiate student-athletes, who often face multiple sources of pressure, including academics, training, competition, and daily life. The presence of these stressors can elicit various stress responses, which in turn may lead to psychological harm among college athletes (Rist et al., 2020). Therefore, understanding and effectively managing the stress response is essential for maintaining the overall health of collegiate student-athletes and preventing long-term negative consequences.

At the same time, driven by the advancement of scientific training concepts, research on the assessment of athletes’ training status has experienced significant growth. Scholars have noted that training induces physiological adaptations, and different training programs exert varying psychological effects on athletes. Training intensity, duration, and frequency have emerged as key focal points in the study of training status, with both physical and psychological dimensions playing crucial roles in athlete development (Chung et al., 2021). Training status refers to the physiological condition of an individual’s body in response to regular physical exercise and structured training. Sustained and systematic training leads to a series of adaptive changes that enhance overall fitness and performance, including improved cardiovascular function, increased muscular strength and endurance, greater flexibility, and enhanced metabolic efficiency (Martinez et al., 2021). The body undergoes specific biochemical and structural modifications to meet the demands of regular exercise, optimizing systems such as the cardiovascular, respiratory, and musculoskeletal systems to better manage the stresses associated with physical activity.

Existing literature confirms a close interconnection between stress response and training status in athletes. An athlete’s stress response is influenced by physiological readiness, training load, and recovery strategies, with stress levels directly impacting training status and subsequent performance outcomes (Lombard, 2021). Furthermore, a comprehensive understanding of the stress response is essential for optimizing the effects of training status on stress management, sport performance strategies, and coping styles among collegiate student-athletes, highlighting the synergistic relationships among these factors (Nuetzel, 2023; Leguizamo Barroso, 2024). Therefore, the aim of this study is to investigate the effects of training status on the stress response of Chinese collegiate student-athletes and to examine the mediating roles of sport performance strategies and coping styles. The findings are intended to inform targeted interventions and strategies for improving training status, optimizing stress responses, enhancing sport performance strategies, and promoting diverse coping styles, thereby providing a scientific basis for advancing training and competition practices among college student-athletes.

## 2 Literature review and research hypotheses

### 2.1 Training status and stress response

Scholars have identified a significant relationship between training status and stress response in athletes. Training status, as a comprehensive indicator encompassing physical function, technical proficiency, and psychological state, plays a pivotal role in determining athletic performance during both training and competition. Concurrently, the stress response—an adaptive reaction to external stimuli—also substantially influences athletes’competitive readiness and psychological wellbeing (Simms et al., 2021; Liu et al., 2021). Research indicates that this relationship is particularly pronounced among collegiate student-athletes, with training status not only directly associated with the type and intensity of stress responses but also serving as a predictor of athletes’ coping abilities and performance levels (Simon et al., 2024).

Moreover, studies have demonstrated a negative correlation between training status and stress response. Specifically, athletes in better training condition tend to exhibit more positive and adaptive stress responses, while those with poorer training status are more susceptible to negative and maladaptive reactions under pressure. This inverse relationship is evident in both physiological markers, such as heart rate variability and cortisol levels, and psychological factors, including anxiety and self-confidence (James et al., 2023). These findings suggest that optimal training status not only enhances physical and technical capacities but also bolsters psychological resilience, enabling athletes to better manage the demands of competition and training. Conversely, the nature of an athlete’s stress response can also impact training outcomes. Positive stress responses may stimulate potential and improve performance, whereas negative responses can lead to diminished condition and an increased risk of injury (Chung et al., 2021; Baskerville et al., 2024). Collectively, these findings underscore a negative correlation between training status and stress response: the better the training status, the lower the level of maladaptive stress response. Therefore, this study proposes Research Hypothesis 1: Training status is a positive predictor of stress response in college student-athletes.

### 2.2 The mediating role of motor performance strategies

One of the key mediating mechanisms examined in this study is the role of sport performance strategies. These strategies, defined as the cognitive and behavioral approaches athletes employ during training and competition, serve as an important link between training status and stress response. Research indicates that a strong training status significantly enhances athletes’ use of effective sport performance strategies (Anshel, 2019). Athletes in good physical and psychological condition are more likely to adopt positive strategies such as goal setting, concentration enhancement, and self-motivation, which facilitate more efficient training and improved competitive performance (Nuetzel, 2023). Furthermore, studies focusing on collegiate student-athletes have shown that those with better training status utilize more targeted and adaptive strategies, enabling them to cope more effectively with complex competitive environments (Vozhzhov et al., 2024). This suggests that training status not only directly influences physical and psychological functioning but also indirectly improves training and competition outcomes by increasing the frequency and quality of sport performance strategy use.

Additionally, there is substantial evidence supporting a significant relationship between sport performance strategies and stress response. The application of these strategies can effectively mitigate negative reactions to stress (Lopes Dos Santos et al., 2020). For instance, experimental research with adolescent shooters demonstrated that positive strategies such as mindfulness training and emotion regulation significantly reduce anxiety and physiological stress responses (Samadi et al., 2021). Moreover, the use of sport performance strategies helps athletes manage stressors and decreases the likelihood of negative emotional and behavioral outcomes arising from academic, training, and competition pressures. This is particularly relevant for collegiate athletes, who face multiple concurrent stressors, making the effective use of such strategies especially critical (Lopes Dos Santos et al., 2020). Thus, sport performance strategies not only alleviate stress responses but also enhance athletes’ mental resilience, allowing for greater adaptability under pressure. Finally, scholars have reiterated that athletes with superior training status are more inclined to adopt positive sport performance strategies, which in turn effectively reduce the intensity and frequency of stress responses. In this way, training status indirectly influences stress response by shaping the selection and application of sport performance strategies (Yang et al., 2024; Bondarchuk et al., 2023). Based on the evidence regarding the complex interplay among training status, sport performance strategies, and stress response, it can be concluded that optimal training status can indirectly decrease stress responses by promoting the use of effective sport performance strategies. Therefore, this study proposes Hypothesis 2: Sport performance strategies mediate the relationship between training status and stress response in college student-athletes.

### 2.3 Mediating role of coping styles

The second mediating mechanism examined in this study is the role of coping styles. Coping styles refer to the cognitive and behavioral strategies individuals employ when confronted with stress or stressors, and their selection is closely linked to the nature of the stress response (Montero-Marin et al., 2014). Research has demonstrated that positive coping styles can significantly reduce both the intensity and frequency of stress responses. For instance, studies involving adolescent athletes have shown that those who utilize problem-focused coping strategies—such as active problem-solving and seeking social support—experience lower levels of anxiety and physiological stress compared to those who rely on emotion-focused coping strategies, such as avoidance and emotional suppression (Samadi et al., 2021). Furthermore, positive coping styles have been found to effectively mitigate negative emotions and behavioral problems among college student-athletes facing the dual pressures of academics and training, thereby lessening the impact of stress on mental health (Cosh and Tully, 2015). These findings underscore the critical role of coping styles in regulating stress responses and highlight the adaptive benefits of positive coping strategies for athletes.

Additionally, scholars have identified training status as a significant factor influencing the choice of coping styles. Athletes in good training condition are more likely to adopt adaptive, problem-focused coping strategies, such as planning, seeking assistance, and adjusting goals, which enable them to manage challenges in training and competition more effectively (Nuetzel, 2023). Those with higher training status tend to demonstrate greater flexibility and adaptability in their coping approaches, allowing them to better balance academic and athletic demands (Leguizamo Barroso, 2024). This suggests that training status not only directly affects athletes’ physical and psychological functioning but also indirectly enhances their capacity to cope with stress by promoting the use of effective coping styles. Finally, research indicates that optimal training status can indirectly reduce athletes’ stress responses by fostering the adoption of positive coping strategies. Athletes with better training status are more inclined to utilize problem-focused coping, which has been shown to effectively diminish the intensity and frequency of stress reactions (Nuetzel, 2023). Based on these findings, this study hypothesizes that coping styles mediate the relationship between training status and stress response; that is, training status influences stress response through its impact on coping styles. Therefore, Hypothesis 3 is proposed: Coping styles mediate the relationship between training status and stress response in college student-athletes.

### 2.4 Chain mediation between motor performance strategies and coping styles

Sport performance strategies and coping styles may jointly serve as a chain mediating mechanism between training status and stress response. Sport performance strategies refer to specific methods and techniques—such as goal setting, concentration, and self-motivation—that athletes use to enhance their performance during training or competition. In contrast, coping styles are the psychological and behavioral responses individuals adopt when facing stress and challenges (Crocker et al., 2015). Research has demonstrated that effective sport performance strategies can significantly improve athletes’ coping abilities. Athletes who employ positive performance strategies are better equipped to regulate their emotions and reduce anxiety under competitive stress, thereby enhancing their overall performance (Robazza et al., 2018). This indicates that sport performance strategies not only influence athletic outcomes but also play a crucial role in stress management. There is a close interaction between sport performance strategies and coping styles, with the former enhancing the effectiveness of the latter, enabling athletes to better manage challenges encountered in training and competition.

Furthermore, scholars have noted that training status encompasses both the psychological and physiological condition of athletes during training. A favorable training status can bolster self-confidence and mental resilience, which subsequently impacts the stress response (Wu et al., 2021). Evidence suggests that well-trained athletes are more likely to adopt positive performance strategies and effective coping styles, and these factors collectively contribute to the regulation of stress responses. Athletes in optimal training condition are able to utilize sport performance strategies more effectively, thereby reducing the intensity of stress responses in high-pressure situations (Low et al., 2023). These findings further support the notion that sport performance strategies and coping styles function as a chain mediating mechanism between training status and stress response. Based on this evidence, the present study hypothesizes that sport performance strategies and coping styles together constitute a chain-mediated pathway through which training status influences stress response. Therefore, Hypothesis 4 is proposed: Sport performance strategies and coping styles jointly mediate the relationship between training status and stress response in college student-athletes.

**Figure 1:**
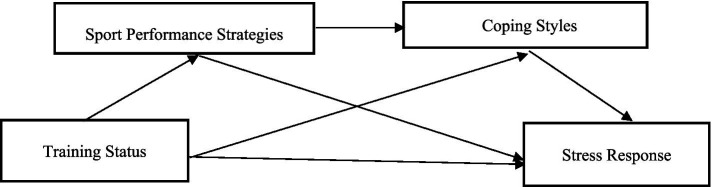
Research framework diagram.

In summary, scholars have highlighted the association between training status and stress response, emphasizing the mediating and chain-mediating roles of sport performance strategies and coping styles within this relationship. These findings provide a solid theoretical foundation for the hypotheses of the present study. Accordingly, the conceptual framework of this research was established (see Figure 1) with the following objectives: (1) to examine the predictive effect of training status on the stress response of college student-athletes; (2) to investigate the mediating role of sport performance strategies in the relationship between training status and stress response; (3) to assess the mediating role of coping styles between training status and stress response; and (4) to explore the chain-mediating effect of sport performance strategies and coping styles in the link between training status and the stress response of college student-athletes.

## 3 Objects and methods of research

### 3.1 Research objects

The Training Status Scale, Stress Response Scale, Sports Performance Strategy Scale, and Coping Style Scale were administered to a random sample of 800 student-athletes from Chinese colleges and universities. Data collection took place in physical education classes, with professionally trained physical education teachers and psychology students serving as the primary administrators. The study received approval from school administrators, sports coaches, and the participants themselves. All questionnaires were completed within 10 min in a group setting, with standardized instructions provided to all subjects. Inclusion criteria prior to questionnaire distribution were as follows: (1) physically and mentally healthy individuals; (2) student-athletes currently enrolled in colleges or universities; and (3) participation in at least three provincial-level competitions. Individuals not meeting these criteria were excluded. After data collection, additional exclusion criteria were applied: (1) missing data; (2) inconsistent responses; and (3) non-collegiate student-athletes. Following these procedures, 797 valid questionnaires were retained for analysis (three were excluded due to missing data or failure to meet inclusion criteria), resulting in a recovery rate of 99.63%. Participants ranged in age from 17 to 27 years (M = 20.31, SD = 2.13), with 442 males (55.5%) and 355 females (44.5%). The sample included 289 international athletes (36.3%), 207 sportsmen (26%), 242 Division I athletes (30.4%), 48 Division II athletes (6%), and 11 Division III athletes (1.4%). No significant differences were found in the main variables across gender, age, or sport level. This study was approved by the Institutional Review Board of Zhaoqing University (2024011). All participants provided written informed consent, which outlined the study’s purpose and procedures, confidentiality assurances, the voluntary nature of participation, and researcher contact information. Additionally, the study controlled for gender, grade, and athletic level as potential confounding variables.

### 3.2 Research methods

#### 3.2.1 Psychometric methods

##### 3.2.1.1 Training status scale

The Athlete Stress-Recovery Questionnaire (Kellmann and Kallus, 2001) and the Training Status Monitoring Scale (TSMS), translated and adapted by Chinese scholar Yannin (Yan, 2003), were used to assess athletes’ training status within the context of Chinese sports culture. The scale comprises 32 items across eight dimensions: emotional stress (e.g., “I feel anxious or depressed”), feeling good (e.g., “I feel as if I can accomplish everything”), fatigue (e.g., “I feel drained”), self-efficacy (e.g., “I am in good physical condition”), self-regulation (e.g., “I will motivate myself to give my best effort during a game or a practice session”), physical recovery (e.g., “I am recovering my strength well”), psychological exhaustion (e.g., “I am bored with the sport I am playing”), and mental exhaustion (e.g., “I am in a bad mood”). Responses are rated on a 5-point Likert scale (1 = strongly disagree, 2 = disagree, 3 = neutral, 4 = agree, 5 = strongly agree), with higher total scores indicating better training status. Previous studies have demonstrated that the Training Status Scale has good reliability and validity for measuring the training status of Chinese college student-athletes (Gao, 2022). In the present study, the scale showed a Cronbach’s alpha coefficient of 0.748, a KMO value of 0.873, and satisfactory fit indices in confirmatory factor analysis, indicating good reliability and validity.

##### 3.2.1.2 Stress response scale

The Stress Response Scale developed by Xian-ming Tan was used to assess the stress response levels of college and university student-athletes (Tan and Chen, 2000). The scale consists of 45 items across six dimensions: interpersonal relationships (e.g., incompatible relationships with teammates), sports injuries (e.g., fear of injuries during training or competitions), competition losses (e.g., impact of losses on training and competitions), environmental factors (e.g., poor training environment affecting progress), daily life events (e.g., the effect of the deaths of family and friends), and internal and external pressures (e.g., serious illness affecting training and competitions). Each item is rated on a 5-point Likert scale (1 = none, 2 = very mild, 3 = moderate, 4 = severe, 5 = extremely severe), with higher total scores indicating greater levels of stress. Previous studies have demonstrated that the Stress Response Scale possesses good reliability and validity for measuring stress responses among Chinese college student-athletes (Liu, 2019; Song, 2021). In the present study, the scale demonstrated a Cronbach’s alpha coefficient of 0.966, a KMO value of 0.919, and satisfactory fit indices in confirmatory factor analysis, indicating excellent reliability and validity.

##### 3.2.1.3 Motor performance strategies scale

The Sports Performance Strategies Scale developed by Thomas et al. (1999) was used to assess athletes’ mental skills and strategies during competition and training. The instrument comprises two subscales: the Competition Strategies Scale (for pre-competition assessment) and the Training Strategies Scale (for routine training assessment). The Competition Strategies Scale includes 32 items across eight dimensions: self-talk (e.g., effective control of self-talk), emotional control (e.g., emotional loss of control under stress), automatization (e.g., performing movements naturally), goal setting (e.g., setting very clear goals), imagery (e.g., mentally rehearsing actions), activation mobilization (e.g., energizing oneself to the optimal level), negative thinking (e.g., imagining oneself regaining composure), and relaxation (e.g., ability to relax when mentally upset). Similarly, the Training Strategies Scale consists of 32 items covering eight dimensions: goal setting (e.g., setting realistic yet challenging goals), emotional control (e.g., feeling annoyed when things do not go well), automatization (e.g., movements and skills appear natural and fluid), relaxation (e.g., using self-relaxation techniques during practice), self-talk (e.g., effective use of self-talk), imagery (e.g., mentally rehearsing movements), attention control (e.g., difficulty maintaining focus during training), and mobilization (e.g., practicing how to energize oneself during training). Both subscales use a 5-point Likert scale (1 = never, 2 = rarely, 3 = sometimes, 4 = often, 5 = always), with higher total scores indicating better sport performance strategies. Previous studies have demonstrated that the Sports Performance Strategies Scale has good reliability and validity for assessing student-athletes in Chinese colleges and universities (Lei, 2016). In the present study, the scale demonstrated a Cronbach’s alpha coefficient of 0.963, a KMO value of 0.911, and satisfactory fit indices in confirmatory factor analysis, indicating excellent reliability and validity.

##### 3.2.1.4 Coping style scale

The Chinese Athletes’ Stress Coping Scale, translated and adapted by Zhong et al. (2004), was used to assess the coping styles of college student-athletes. The scale comprises 24 items across four dimensions: problem solving (e.g., solving problems step by step), emotional coping (e.g., trying to calm down), avoidance (e.g., taking a step back to find a better place to live), and transcendental coping (e.g., letting everything take its course). Each item is rated on a 5-point Likert scale (1 = never, 2 = occasionally, 3 = sometimes, 4 = often, 5 = always), with higher total scores indicating more adaptive coping styles. Previous studies have demonstrated that this scale possesses good reliability and validity for measuring coping styles among student-athletes in Chinese colleges and universities (Ning, 2022). In the present study, the scale showed a Cronbach’s alpha coefficient of 0.953, a KMO value of 0.893, and satisfactory fit indices in confirmatory factor analysis, indicating excellent reliability and validity.

#### 3.2.2 Mathematical and statistical methods

The data in this study were statistically analyzed using SPSS 21.0, with the PROCESS macro developed by Hayes. First, descriptive statistics and difference tests (*p* < 0.05) were conducted on demographic information, training status, stress response, sport performance strategies, and coping styles. Second, common method bias was assessed using Harman’s single-factor test in SPSS 26.0. Third, Pearson’s correlation analysis was performed to examine the bivariate relationships among training status, stress response, sport performance strategies, and coping styles in college student-athletes. Fourth, the independent mediating effects of sport performance strategies and coping styles, as well as their chain mediating effect between training status and stress response, were tested using PROCESS macro Model 6 and the Bootstrap resampling technique (5,000 iterations). Statistical significance was set at *p* < 0.05 for all analyses.

## 4 Results

### 4.1 Descriptive statistics of training status, stress response, sports performance strategies, and coping styles

The statistical results presented in Table 1 indicate that both training status and sport performance strategies showed significant differences across age, gender, and sport level (*p* < 0.05). Stress response demonstrated significant differences by age and sport level (*p* < 0.05), but not by gender (*p* > 0.05). Coping style was significantly different only across sport levels (*p* < 0.05), with no significant differences observed for gender or age (*p* > 0.05). Male athletes had higher mean scores than female athletes in training status, stress response, and sport performance strategy, while both genders had similar scores for coping style. Additionally, the four variables exhibited distinct patterns across different statistical categories, providing further insight into the influence and interrelationships among training status, sport performance strategy, coping style, and stress response in this study.

**Table 1:** Descriptive statistics of training status, stress response, motor performance strategies and coping style test results (M ± SD).

Sex	*N*/people	Training status	Stress response	Sport performance strategies	Coping styles
Male	442	90.04 ± 11.03	75.57 ± 28.50	209.46 ± 35.31	83.73 ± 16.63
Female	355	86.25 ± 10.58	73.03 ± 23.45	203.451 ± 29.27	83.73 ± 15.17
Overall	797	88.36 ± 10.99	74.44 ± 26.38	206.78 ± 32.88	83.73 ± 15.99
Gender difference (T/p)		4.91/0.00	1.35/0.18	2.57/0.01	0.01/0.99
Age difference (F/p)		2.26/0.00	3.29/0.00	3.11/0.00	1.58/0.09
Difference in sport level (F/p)		16.31/0.00	5.22/0.00	5.34/0.00	4.01/0.00

*P* < 0.05 represents significant differences in variables between gender, age, and sport level.

### 4.2 Common methodological bias test

The use of questionnaires for data collection may introduce the risk of common method bias. To address this, Harman’s one-factor test was conducted prior to data analysis for statistical control, whereby all items from the study variables were subjected to unrotated principal component factor analysis (Tehseen et al., 2017). The results indicated that 25 factors with eigenvalues >1 were extracted, and the variance explained by the first factor was 26.22%, which is below the critical threshold of 40%. This suggests that no single factor accounted for the majority of the variance, indicating that common method bias was not a serious concern in this study. Therefore, the data were deemed suitable for subsequent chain mediation effect analysis.

### 4.3 Correlation analysis of training status, stress response, sports performance strategy and coping style

As shown in Table 2, training status was significantly negatively correlated with stress response, and significantly positively correlated with both sport performance strategy and coping style. Additionally, stress response was significantly negatively correlated with sport performance strategy and coping style, while sport performance strategy was significantly positively correlated with coping style. These correlations among the variables provide empirical support for the subsequent hypothesis testing and establish a solid foundation for the mediation effect analysis in this study.

**Table 2:** Correlation analysis statistics of training status, stress response, sports performance strategies and coping styles.

Variable	M ± SD	Training status	Stress response	Sport performance strategies	Coping styles
Training status	88.36 ± 10.99	1			
Stress response	74.44 ± 26.38	−0.679**	1		
Sport performance strategies	206.78 ± 32.88	0.640**	−0.615**	1	
Coping styles	83.73 ± 15.99	0.571**	−0.643**	0.733**	1

*N* = 797. *p < 0.05, **p < 0.01. Same below.

### 4.4 Test of mediating effects of sport performance strategies and coping styles

**Figure 2:**
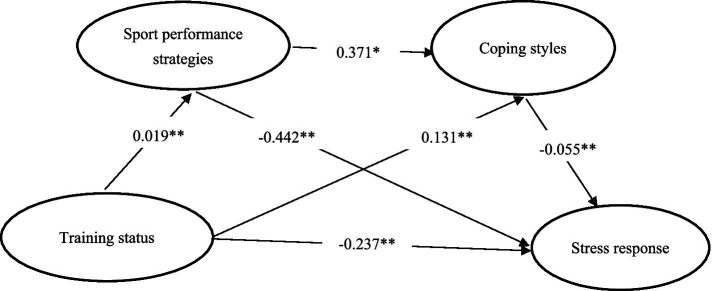
Chain mediation model diagram of sport performance strategies and coping styles between training state and stress response.

Using training status as the independent variable, sport performance strategies and coping styles as mediators, and stress response as the dependent variable, the PROCESS macro for SPSS developed by Hayes was employed, configured according to Model 6. The bias-corrected nonparametric percentile Bootstrap method (5,000 resamples) was used to calculate 95% confidence intervals for the effects in the chain mediation model (Wu et al., 2024). As shown in Table 3 and the path coefficient diagram (Figure 2), the total effect of training status on stress response among college student-athletes was −0.519 (t = 11.541, *p* < 0.001). The direct path coefficient from training status to stress response was −0.237 (t = −13.539, *p* < 0.001). The path coefficient from training status to sport performance strategies was 0.019 (t = 10.211, *p* < 0.001), and from sport performance strategy to stress response was −0.442 (t = −5.879, *p* < 0.001). The path coefficient from training status to coping styles was 0.131 (t = 3.495, *p* < 0.001), and from coping styles to stress response was −0.055 (t = −1.435, *p* < 0.001). Additionally, the path coefficient from sport performance strategy to coping style was 0.371 (t = 29.986, *p* < 0.001). All path coefficients reached statistical significance (*p* < 0.001). These results provide empirical support for research hypothesis H1.

**Table 3:** Regression analysis of the chain-mediated model between training status and stress response.

Variable	Sport performance strategies		Coping styles		Stress response		Aggregate Effect	
	*β*	*t*	*β*	*t*	*β*	*t*	*β*	*t*
Training status	0.019	10.211**	0.131	3.495**	−0.237	−13.539**	−0.519	11.541**
Sport performance strategies			0.371	29.986**	−0.442	−5.879**		
Coping styles					−0.055	−1.435**		
R^2^	0.116		0.545		0.493		0.144	
F	104.259**		474.763**		84.883**		133.211**	

*p < 0.05, **p < 0.01.

After conducting the standardized effect size and significance tests for the pathways by which training status influences the stress response of college student-athletes, the results indicate that the Bootstrap 95% confidence interval for the total indirect effect does not include zero. This demonstrates a significant mediating effect of both sport performance strategy and coping style between training status and stress response (total indirect effect = −0.282, accounting for 54.33% of the total effect). The mediating effect comprises three specific indirect pathways:(1) The indirect effect through the “training status → sport performance strategy → stress response” pathway has a Bootstrap 95% confidence interval that does not include zero, indicating that sport performance strategy serves as a significant mediator between training status and stress response (standardized effect = −0.056, accounting for 10.79% of the total effect), thus supporting Hypothesis H2;(2) The indirect effect through the “training status → coping style → stress response” pathway also has a Bootstrap 95% confidence interval excluding zero, indicating that coping style significantly mediates the relationship between training status and stress response (standardized effect = −0.168, accounting for 32.37% of the total effect), thereby confirming Hypothesis H3;(3) The indirect effect via the “training status → sport performance strategy → coping style → stress response” pathway is significant, as the Bootstrap 95% confidence interval does not include zero. This demonstrates that sport performance strategy and coping style together play a significant chain mediating role between training status and stress response (standardized effect = −0.058, accounting for 11.18% of the total effect), thus verifying Hypothesis H4. These findings collectively confirm the significant mediating and chain mediating roles of sport performance strategy and coping style in the relationship between training status and stress response among college student-athletes (Table 4).

**Table 4:** Chain mediation effect test of sport performance strategies and coping styles on training status and stress response.

Type of effect	Efficiency value	Boot SE	Bootstrap 95% CI		Efficiency ratio
			lower limit	Upper limit	
Total effect	−0.519	0.079	0.755	1.064	100%
Direct effect	−0.237	0.081	0.921	1.232	45.66%
Training status - sport performance strategies - stress response	−0.056	0.048	−0.155	−0.037	10.79%
Training status - coping styles - stress response	−0.168	0.035	−0.245	−0.108	32.37%
Training status - sport performance strategies - coping styles - stress response	−0.058	0.017	0.029	0.095	11.18%
Total indirect effect	−0.282	0.052	−0.278	−0.072	54.33%

## 5 Discussion

### 5.1 Training status and stress response

The results of this study showed that training status had a significant negative effect on the stress response of college student-athletes; that is, better training status was associated with lower stress response, confirming Hypothesis 1. This finding is consistent with previous research. Physiological studies have demonstrated that regular physical activity and higher fitness levels are strongly linked to a more effective and adaptive stress response system. Individuals with greater aerobic fitness exhibit a weaker cortisol response to acute stress, indicating more controllable and adaptive stress regulation (Moyers and Hagger, 2023). This physiological adaptation underlies the negative correlation between training status and stress response. Additionally, research has found that those who regularly engage in physical activity report lower perceived stress and higher overall wellbeing (Kisker and Schöne, 2024), further supporting these results. From a psychological perspective, physical activity is associated with improved mood, reduced anxiety, and enhanced coping mechanisms, partly due to the release of endorphins, which help alleviate stress (Hossain et al., 2024). The “fitness-fatigue model” suggests that while exercise induces short-term fatigue and an acute stress response, long-term training leads to physiological adaptations that reduce stress responses during both exercise and recovery (Stephens Hemingway, 2021; Chiu and Barnes, 2003). In addition, an increasing number of functional studies in recent years have utilized neuroimaging techniques such as electroencephalography (EEG) and magnetic resonance imaging (MRI) to further elucidate the relationship between exercise training and stress responses. For example, EEG studies have found that systematically trained athletes exhibit increased alpha wave activity in prefrontal regions when confronted with stressful situations, which is closely associated with improved emotion regulation and stress adaptation (Petruzzello et al., 1991; Li et al., 2024). MRI research has also demonstrated that regular exercise training induces structural and functional changes in brain regions involved in emotion regulation, such as the prefrontal cortex and amygdala, including increased gray matter volume and enhanced neural network connectivity (Erickson et al., 2011; Wu et al., 2022). These experimental intervention findings suggest that a good training status not only leads to physiological and psychological adaptations, but also enhances the brain’s capacity for stress regulation through neuroplastic mechanisms. This provides a mechanistic explanation for the negative correlation between training status and stress response observed in the present study. Therefore, integrating evidence from functional experimental studies such as EEG and MRI can offer deeper insights into how exercise training enhances the stress adaptation capacity of college athletes by improving brain function and structure. As can be seen, the above findings not only support the findings in the existing literature, but also emphasize the potential protective effect of well-managed training on mental health in college athletes.

Furthermore, the research and test data from this study indicate significant differences in training status, stress response, sport performance strategies, and coping styles among college student-athletes based on age and gender. The mean scores for each scale were higher for male students compared to female students, which is consistent with previous research findings. According to social role theory, societal expectations regarding gender roles influence the behaviors and performance of males and females in various contexts. Males are typically encouraged to display greater competitiveness and self-confidence in physical activities, which may contribute to higher scores in training status and sport performance strategies. In contrast, females often face additional social role pressures, such as balancing academic and athletic responsibilities, which may impact their training status and coping styles (Gong et al., 2021). Moreover, studies have shown that males generally possess higher muscle mass and cardiorespiratory fitness, enabling better performance in physical training. Males are also more likely to adopt problem-focused coping strategies when facing stress, whereas females may be more inclined toward emotion-focused strategies, potentially leading to differences in stress responses (Li and Zhao, 2024; Hong and Hong, 2024). For instance, one study found that male athletes were more likely to cope with failure by increasing training intensity, while female athletes tended to seek social support (Hong and Hong, 2024; Gong et al., 2021). However, not all variables exhibited gender differences, which may be attributed to individual differences and environmental factors. Some female athletes may achieve comparable performance levels to their male counterparts through individualized training and psychological support strategies. Therefore, understanding these gender differences is essential for developing more inclusive training and support programs that enable all athletes to achieve optimal performance in both sport and mental health.

### 5.2 The mediating role of motor performance strategies

The results of this study demonstrated that sport performance strategy mediates the relationship between training status and stress response among college student-athletes, thus confirming Hypothesis 2. This finding aligns with previous research. Studies have shown that sport performance strategies, such as goal setting and visualization, effectively alleviate athletes’ stress responses by enhancing their sense of control and self-confidence, thereby reducing perceived threat and stress in challenging situations (Nuetzel, 2023). These strategies help athletes manage emotions and maintain focus, leading to calmer performance under pressure. Furthermore, research indicates that higher training levels motivate athletes to develop and implement more effective sport performance strategies. Elite athletes, due to their extensive training and experience, are more likely to employ complex strategies, which not only support physical progress but also provide psychological resilience against training-related stress (Vozhzhov et al., 2024). As training intensity increases, athletes receive more mental skills training, further promoting the use of advanced sport performance strategies (Lopes Dos Santos et al., 2020). For example, athletes who utilize goal-setting and self-talk demonstrate more adaptive stress responses during high-intensity training phases (Anshel, 2019). Overall, these findings underscore the crucial mediating role of sport performance strategies in the relationship between training status and stress response, highlighting the importance of developing these strategies to optimize psychological outcomes in college athletes.

### 5.3 Mediating role of coping styles

This study also found that coping styles mediate the relationship between training status and stress response among college student-athletes, thus confirming Hypothesis 3. This result is consistent with previous research. According to the stress and coping transaction model, individuals’ coping strategies largely determine their responses to stressors, with adaptive coping styles-such as problem-focused coping and positive reappraisal-effectively reducing stress responses (Bondarchuk et al., 2024). Athletes who employ positive coping strategies experience lower stress levels during high-intensity training, as these strategies help redefine stressors and enhance control and self-confidence, thereby mitigating negative stress effects (Ma, 2024). Furthermore, research indicates that higher training status encourages the development of more effective coping strategies. Athletes in better training condition typically exhibit more adaptive coping styles, and elite athletes are more likely to use positive self-talk and goal setting to manage training-related stress (Cosh and Tully, 2015; Lopes Dos Santos et al., 2020). High training demands require athletes to continually develop effective coping mechanisms. As training conditions improve, athletes are more likely to adopt adaptive coping styles, which buffer the impact of stressors and reduce stress response intensity (Montero-Marin et al., 2014). For example, athletes accustomed to high-intensity training may use problem-centered coping to break down challenges, resulting in better stress management in competitive settings (Zschucke et al., 2015). These findings highlight the importance of fostering adaptive coping styles through targeted training, as this can reduce stress responses and enhance both athletic performance and mental health.

### 5.4 Chain mediation between motor performance strategies and coping styles

Based on these findings, this study also demonstrated that sport performance strategies and coping styles jointly serve as a chain mediator between training status and stress response among college student-athletes, thus confirming Hypothesis 4. This result is consistent with previous research. Studies have shown that sport performance strategies and coping styles are complementary in athletes’ psychological development (Crocker et al., 2015; Robazza et al., 2018). According to the Stress and Coping Transactional Model, coping styles represent the cognitive and behavioral efforts individuals use to manage stress, while sport performance strategies are specific techniques athletes employ to optimize performance. Athletes who utilize goal-setting strategies are more likely to adopt problem-focused coping in high-pressure situations, demonstrating a synergistic effect between the two. Effective strategies such as goal setting, positive self-talk, and visualization align with adaptive coping, equipping athletes with a comprehensive mental toolkit to address various stressors (Devonport, 2015). Furthermore, research indicates that athletes with higher training status often participate in systematic mental skills training, incorporating techniques like visualization and self-talk into their routines. These athletes demonstrate superior mental skills, enabling them to manage stress more effectively during competition (Nuetzel, 2023; Leguizamo Barroso, 2024). As training status improves, athletes not only refine physical abilities but also develop more adaptive coping styles, which help reduce stress and anxiety (McLoughlin et al., 2021). In summary, higher training status facilitates the use of sport performance strategies through mental skills training, which in turn promotes adaptive coping styles that buffer stress responses. This chain-mediated effect underscores the importance of integrating mental skills training and coping strategies in athlete development. The results highlight that enhancing sport performance strategies and coping styles is crucial for helping collegiate athletes effectively manage stress.

## 6 Practical significance

This study provides a valuable theoretical foundation and practical guidance for enhancing college athletes’ sport learning outcomes. By elucidating the mechanisms through which training status, sport performance strategies, and coping styles influence stress responses, the findings offer a basis for coaches, trainers, and sport psychologists to design individualized training programs. Such programs should not only emphasize physical performance but also incorporate strategies to strengthen coping skills and optimize stress responses, thereby supporting both athletic performance and psychological wellbeing. Furthermore, these results highlight directions for future research, encouraging studies with more diverse populations and the use of multiple methodologies to deepen understanding of the complex interplay between training, psychological factors, and athletic performance.

## 7 Research shortcomings and outlook

This study provides important insights into the relationship between training status and stress response among Chinese college athletes; however, several limitations should be acknowledged. First, the specificity of the sample is a notable limitation. As the study focused solely on Chinese college athletes, the findings may not be generalizable to athletes from other cultural backgrounds or age groups. Cultural differences can influence psychological and behavioral responses, and thus, the applicability of these results to cross-cultural contexts remains to be validated. Additionally, the reliance on self-report measures, while efficient for large-scale data collection, may introduce social desirability and recall biases, potentially affecting data accuracy and reliability. Self-report methods may also fail to capture the complexity of psychological variables, particularly subtle emotional and behavioral differences. Finally, the study design did not fully account for external factors such as social support, cultural background, and economic status, which may moderate or mediate the relationship between training status and stress response.

Future research should address these limitations to enhance the robustness and applicability of the findings. Expanding the sample to include athletes from diverse regions, cultural backgrounds, and age groups would help validate the generalizability and cross-cultural relevance of the results. Employing multiple assessment methods—such as observational measures, physiological indicators (e.g., heart rate variability, cortisol levels), and qualitative interviews—would provide more comprehensive and objective data, reducing the limitations of self-report. Furthermore, future studies should systematically consider additional external variables, including social support, cultural factors, economic status, and individual differences, to gain a deeper understanding of the complex interplay between training status, psychological factors, and stress response. These improvements will contribute to the development of more effective interventions to enhance athletes’ performance and psychological wellbeing, and provide a more comprehensive theoretical framework for the field of sport psychology.

## 8 Conclusion

This study found significant gender differences in training status, stress response, sport performance strategies, and coping styles among collegiate student-athletes, with males outperforming females in mean scores across all variables. More importantly, training status emerged as a key predictor of sport performance strategies, coping styles, and stress responses. Specifically, better training status not only directly enhanced athletes’ sport performance strategies but also indirectly improved stress responses through more adaptive coping styles. These findings highlight the mediating and moderating roles of training status in the psychological state and athletic performance of college student-athletes. Future research should further investigate the specific mechanisms by which different training states influence athletes’ psychological and physiological outcomes, as well as strategies to optimize training status for enhanced overall performance. The results of this study provide a theoretical reference for understanding the relationship between psychological state and athletic performance in college student-athletes.

## 9 Recommendations

Based on the results and analyses of this study, three recommendations are proposed to address the improvement of training status and stress response in college athletes:

1. Individualized training plans: coaches and trainers should develop individualized training plans based on athletes’ individual characteristics (e.g., gender, age, and cultural background). These programs should not only focus on physical performance, but should also integrate mental training strategies to improve the athlete’s coping skills and optimize his/her stress response. By regularly evaluating and adjusting the training programs, the individual needs of athletes can be better met and their overall performance can be enhanced.
2. Multi-dimensional assessment and feedback mechanism: it is recommended that multi-dimensional assessment methods be introduced into the training process, including physiological indicators (e.g., heart rate, cortisol level) and psychological assessments (e.g., stress tests, emotional state surveys). These assessments can provide more comprehensive feedback to help coaches and athletes identify potential stressors and the effectiveness of coping strategies. Through regular feedback and adjustments, the training status and mental health of athletes can be managed more effectively.
3. Strengthen social support and psychological counseling: establish a supportive training environment to encourage mutual assistance and teamwork among athletes. At the same time, provide professional psychological counseling services to help athletes better understand and manage their emotions and stress. Through regular psychological counseling and team building activities, athletes’ mental toughness can be enhanced and their ability to perform under high-pressure environments can be improved.

## Data Availability

The datasets presented in this article are not readily available because the study involved athletes, a confidentiality agreement was signed when the questionnaire was administered, so the raw data for the thesis could not be made public and was used only for the writing of the thesis. Requests to access the datasets should be directed to HL, 1317463029@qq.com.
