# Supplementary material for: Relationship between training status and stress response in Chinese college student-athletes: chain mediation between sport performance strategies and coping styles
Source: Front Psychol. 2025 Jul 9;16:1597539. doi: 10.3389/fpsyg.2025.1597539 (PMC12285528; doi:10.3389/fpsyg.2025.1597539)
Supplement: Supplementary file 1 [file Data_Sheet_1.zip › Data Sheet_1/scale/Athlete Stress Scale.docx]

Athlete Stress Scale

The following statements are all likely to happen to athletes in daily life, training and competition. Please fill in the form according to your actual situation in the last 3 months (1=None, 2=Very mild, 3=Moderate, 4=Severe, 5=Extremely severe) There is no right or wrong answer so please answer truthfully and ask if you still have any questions.

No Very light Moderate Heavier Severe

1. fear of injury when training or competing 1 2 3 4 5

2. I do not feel good in an unfamiliar playing field 1 2 3 4 5

3. being misunderstood or wrongly accused 1 2 3 4 5

4. the impact of personal financial difficulties on oneself 1 2 3 4 5

5. Feeling unwell 1 2 3 4 5

6. severe insomnia affecting training and competition 1 2 3 4 5

7. fear of unfair refereeing during matches 1 2 3 4 5

8. Injury during training or competition 1 2 3 4 5

9. Coach always criticizes you 1 2 3 4 5

10. I have trouble adapting to the competition environment 1 2 3 4 5

11. serious illness of a family member worries me 1 2 3 4 5

12. I get distracted during games 1 2 3 4 5

13. I have problems with my coach 1 2 3 4 5

14. poor training environment affecting my progress 1 2 3 4 5

15. the effect of being involved in a civil legal dispute on oneself 1 2 3 4 5

16. Being overly nervous during competitions 1 2 3 4 5

17. too much public pressure 1 2 3 4 5

18. Shock of an accident 1 2 3 4 5

19. can't get rid of the shadow of previous injuries 1 2 3 4 5

20. being punished 1 2 3 4 5

21. feeling bad about going away to play in a foreign country 1 2 3 4 5

22. feeling like you have no one to talk to 1 2 3 4 5

23. Loss of sports affects training and competition 1 2 3 4 5

24. impact of high debt on self 1 2 3 4 5

25. Thinking too much about competition results 1 2 3 4 5

26. being devastated by a breakup 1 2 3 4 5

27. the effect of family disharmony on oneself 1 2 3 4 5

28. Lack of desire to win in competitions 1 2 3 4 5

29. Sudden loss of self-confidence on the field 1 2 3 4 5

30. Poor relationship with teammates 1 2 3 4 5

31. Recurrence of an old injury that bothers you 1 2 3 4 5

32. the impact of death of a family member or friend 1 2 3 4 5

33. Feeling unappetizing before a match 1 2 3 4 5

34. worrying about my future 1 2 3 4 5

35. being scared when thinking about other people's serious injuries 1 2 3 4 5

36. serious illness affects training and competition 1 2 3 4 5
