# Supplementary material for: Relationship between training status and stress response in Chinese college student-athletes: chain mediation between sport performance strategies and coping styles
Source: Front Psychol. 2025 Jul 9;16:1597539. doi: 10.3389/fpsyg.2025.1597539 (PMC12285528; doi:10.3389/fpsyg.2025.1597539)
Supplement: Supplementary file 1 [file Data_Sheet_1.zip › Data Sheet_1/scale/Athlete Training Status Monitoring Scale.docx]

Athlete Training Status Monitoring Scale

The purpose of this measure is to know your mental state, physical health and activity during the past 3 days (including day and night), and we regret that there are five options for each question. There are five options for each question, which represent the frequency of each situation. Please circle the option that best reflects your mental and physical activity.

1、I feel anxious or depressed 1 2 3 4 5

2、I am in a good mood 1 2 3 4 5

3、I feel drained 1 2 3 4 5

4. I feel as if I can accomplish everything 1 2 3 4 5

5. I motivate myself before a game or practice 1 2 3 4 5

6. I am in good physical condition 1 2 3 4 5

7. I feel like quitting the sport 1 2 3 4 5

8. I feel unresponsive when training or competing 1 2 3 4 5

9、I feel upset 1 2 3 4 5

10、I feel happy 1 2 3 4 5

11. I feel tired 1 2 3 4 5

12. I am sure I am doing well 1 2 3 4 5

13. I motivate myself to give my best during a game or practice 1 2 3 4 5

14. I feel energized 1 2 3 4 5

15. I feel that training and competitions are meaningless to me 1 2 3 4 5

I feel that the accuracy and smoothness of my technique decreases during training or competition 1 2 3 4 5

17. I feel depressed 1 2 3 4 5

18. I feel happy 1 2 3 4 5

19. I feel back pain 1 2 3 4 5

20. Everything I do is successful 1 2 3 4 5

21. I motivate myself to get into shape as soon as possible when practicing or competing 1 2 3 4 5

22. I recover my strength well 1 2 3 4 5

23. I get bored with my sport - 1 2 3 4 5

24. I feel less flexible and less coordinated during training or competitions 1 2 3 4 5

25. I am in a bad mood 1 2 3 4 5

26. I feel relaxed 1 2 3 4 5

27. I have aches and pains all over my body 1 2 3 4 5

28. I am sure that I can play at my own level at any time 1 2 3 4 5

29. When I made a mistake, I adjusted my mind and continued to play 1 2 3 4 5

30. I feel in good physical condition 1 2 3 4 5

31. I find it difficult to continue my training 1 2 3 4 5

32. I feel very inefficient in training 1 2 3 4 5
