# Supplementary material for: Relationship between training status and stress response in Chinese college student-athletes: chain mediation between sport performance strategies and coping styles
Source: Front Psychol. 2025 Jul 9;16:1597539. doi: 10.3389/fpsyg.2025.1597539 (PMC12285528; doi:10.3389/fpsyg.2025.1597539)
Supplement: Supplementary file 1 [file Data_Sheet_1.zip › Data Sheet_1/scale/Chinese Athletes Coping Scale.docx]

Chinese Athletes Coping Scale

Guideline: The following statements describe some of the things you may have done or thought when you encountered stress in training and competitions in the recent year, please read them carefully and put a tick on the corresponding number according to the extent to which the content of the sentence corresponds to your actual situation. 1 stands for “never like this”, 5 stands for “always like this”, and the rest of the numbers are in between. 1 is “never like this”, 5 is “always like this”, and the rest of the numbers are in between.

The closer the number is to 1, the closer it is to “never”, and the closer the number is to 5, the closer it is to “always”.

Step by step to solve the problem 1 2 3 4 5

2. Use positive mental skills to relieve stress 1 2 3 4 5

3 Think about something I like 1 2 3 4 5

4 Concentrate on what needs to be done 1 2 3 4 5

5 Try harder 1 2 3 4 5

6 Wait and see what happens 1 2 3 4 5

7 Analyze the problem and find a way to deal with it 1 2 3 4 5

8 Make a plan of action to overcome the problem and follow it 1 2 3 4 5

9 Try to calm yourself down 1 2 3 4 5

10 Leave a stressful situation or environment 1 2 3 4 5

11 Do something else that I enjoy 1 2 3 4 5

12 Not thinking about stressful situations 1 2 3 4 5

13 I will return to my normal routine 1 2 3 4 5

14 Minimize my emotional reactions 1 2 3 4 5

15 Keep my mood light and happy 1 2 3 4 5

16 Try to be in a good mood 1 2 3 4 5

17 Take a step back 1 2 3 4 5

18 Find ways to control my emotions 1 2 3 4 5

19 Take a vacation to make myself forget the difficulties 1 2 3 4 5

20 Winning and losing is a matter of course 1 2 3 4 5

21 Not facing stressful problems 1 2 3 4 5

22 Follow my destiny (I deserve it) 1 2 3 4 5

23 Keep your emotions under control 1 2 3 4 5

24 Let things take their own course and go with the flow 1 2 3 4 5
