# Supplementary material for: Relationship between training status and stress response in Chinese college student-athletes: chain mediation between sport performance strategies and coping styles
Source: Front Psychol. 2025 Jul 9;16:1597539. doi: 10.3389/fpsyg.2025.1597539 (PMC12285528; doi:10.3389/fpsyg.2025.1597539)
Supplement: Supplementary file 1 [file Data_Sheet_1.zip › Data Sheet_1/scale/Sports Performance Strategies Scale.doc]

Sports Performance Strategies Scale

1. Competition Strategy Scale

Where: 1=never, 2=very rarely, 3=sometimes, 4=frequently, 5=always present.

1 Energize yourself to be at just the right level 1 2 3 4 5

2 Imagine yourself revitalized 1 2 3 4 5

3 Imagine in your mind that the game is going the way you want it to.

1 2 3 4 5

4 Preview the action in your mind 1 2 3 4 5

5 I can relax when I feel uneasy 1 2 3 4 5

6 I can control my self-talk effectively 1 2 3 4 5

7 Previewed in my imagination what it would feel like to do the action 1 2 3 4 5

8 Setting very clear goals 1 2 3 4 5

9 Saying cues that are relevant to your better game 1 2 3 4 5

10 When the body is so tense that the muscles are stiff, find it

find it hard to relax 1 2 3 4 5

11 Positively saying things that will enable you to perform at your best level of competition 1 2 2 4 5

1 2 3 4 5 11 Positive things to say to help you perform at your best during a race 1 2 3 4 5

12 Lost control of emotions under pressure 1 2 3 4 5

13 Has set personal performance goals 1 2 3 4 5

14 Knows how to reduce stress when under pressure 1 2 3 4 5

15 Evaluates whether he/she has achieved race goals 1 2 3 4 5

16 Got myself excited and ready to compete 1 2 3 4 5

17 When something upsets me and affects my performance 1 2 3 4 5

18 Raising my energy level when necessary 1 2 3 4 5

19 Thinking about failure 1 2 3 4 5

20 Make movements very naturally 1 2 3 4 5

21 Setting clear goals for results 1 2 3 4 5

22 Saying specific cues to help yourself race 1 2 3 4 5

23 Self-talk is very negative 1 2 3 4 5

24 I am able to relax if needed to prepare for the race 1 2 3 4 5

25 Without a little conscious effort, the action is completed as if it

It is an instinctive behavior 1 2 3 4 5

26 Bad moods prevent me from performing at my best in competition 1 2 3 4 5

27 Keep thinking positive thoughts 1 2 3 4 5

28 Doing something to cheer myself up 1 2 3 4 5

29 Making a mistake makes it difficult to refocus my attention

on the task at hand 1 2 3 4 5

30 Not thinking about the movement too much, just letting it happen 1 2 3 4 5

31 Doing a movement without my brain consciously thinking about how it should be done 1 2 3 4 5

32 Visualize the movement in your mind before the match 1 2 3 4 5

2.Training Strategies Scale

Where: 1=never, 2=very rarely, 3=sometimes, 4=frequently, 5=always present.

1 Use self-talk effectively 1 2 3 4 5

2 Practice a method of relaxation 1 2 3 4 5

3 Movements and skills look natural and fluid 1 2 3 4 5

4 Say something to yourself to help improve your training performance 1 2 3 4 5

5 Feel annoyed when things are going badly 1 2 3 4 5

6 The whole skill and movement happens naturally, without needing to be yourself

Attention to each part of the skill 1 2 3 4 5

7 Difficulty energizing myself in teaching tests 1 2 3 4 5

8 Inability to concentrate during training 1 2 3 4 5

9 To help myself utilize my training time more effectively

Setting goals 1 2 3 4 5

10 I am able to control distracting thoughts during training 1 2 3 4 5

11 It is difficult to motivate myself when I am not in high spirits 1 2 3 4 5

12 Imagine successful actions from the past 1 2 3 4 5

13 Motivate myself to train through positive self-talk 1 2 3 4 5

14 Using Relaxation Techniques in Teaching Quiz Sessions 1 2 3 4 5

15 Learn how to motivate yourself during training 1 2 3 4 5

16 Don't set goals for training, just train 1 2 3 4 5

17 When things get bad. I can't control my emotions 1 2 3 4 5

18 Use training time to practice relaxation techniques 1 2 3 4 5

19 Feeling discouraged and upset when training does not go well 1 2 3 4 5

I feel discouraged and upset 1 2 3 4 5

20 Rehearse your movements in your head 1 2 3 4 5

21 Not thinking too much about the movement, just letting it happen 1 2 3 4 5

22 Relax yourself during training 1 2 3 4 5

23 Very specific goals 1 2 3 4 5

24 Set realistic and challenging goals 1 2 3 4 5

25 Difficulty in maintaining concentration throughout long training sessions 1 2 3 4 5

26 Maximize the effect you can achieve in training by saying positive things 1 2 3 4 5

27 Practicing how to energize yourself during training sessions 1 2 3 4 5

28 During training sessions, everything goes smoothly 1 2 3 4 5

29 I can't concentrate when I move badly 1 2 3 4 5

30 When I visualize my movements, I imagine all kinds of feelings at the time 1 2 3 4 5

31 Concentrate effectively 1 2 3 4 5

32 When I visualize my movements, it is like watching a movie. I watch myself on the screen as if I were watching a movie 1 2 3 4 5
